# Kinetics of Aggregation and Magnetic Separation of Multicore Iron Oxide Nanoparticles: Effect of the Grafted Layer Thickness

**DOI:** 10.3390/nano8080623

**Published:** 2018-08-17

**Authors:** Hinda Ezzaier, Jéssica Alves Marins, Cyrille Claudet, Gauvin Hemery, Olivier Sandre, Pavel Kuzhir

**Affiliations:** 1 CNRS UMR 7010 Institute of Physics of Nice (INPHYNI), University Côte d’Azur, Parc Valrose, 06108 Nice, France; ezzaier.hinda@gmail.com (H.E.); jessica.amarins@gmail.com (J.A.M.); cyrille.claudet@unice.fr (C.C.); 2 Laboratory of Physics of Lamellar Materials and Hybrid Nano-Materials, Faculty of Sciences of Bizerte, University of Carthage, Zarzouna 7021, Tunisia; 3 CNRS UMR 5629, Laboratoire de Chimie des Polymères Organiques (LCPO), University of Bordeaux, ENSCBP 16 Avenue Pey Berland, 33607 Pessac, France; gauvin.hemery@gmail.com (G.H.); olivier.sandre@enscbp.fr (O.S.)

**Keywords:** iron oxide nanoparticles, nanoflowers, magnetic separation, grafted layer

## Abstract

In this work, we have studied field-induced aggregation and magnetic separation—realized in a microfluidic channel equipped with a single magnetizable micropillar—of multicore iron oxide nanoparticles (IONPs) also called “nanoflowers” of an average size of 27 ± 4 nm and covered by either a citrate or polyethylene (PEG) monolayer having a thickness of 0.2–1 nm and 3.4–7.8 nm, respectively. The thickness of the adsorbed molecular layer is shown to strongly affect the magnetic dipolar coupling parameter because thicker molecular layers result in larger separation distances between nanoparticle metal oxide multicores thus decreasing dipolar magnetic forces between them. This simple geometrical constraint effect leads to the following important features related to the aggregation and magnetic separation processes: (a) Thinner citrate layer on the IONP surface promotes faster and stronger field-induced aggregation resulting in longer and thicker bulk needle-like aggregates as compared to those obtained with a thicker PEG layer; (b) A stronger aggregation of citrated IONPs leads to an enhanced retention capacity of these IONPs by a magnetized micropillar during magnetic separation. However, the capture efficiency Λ at the beginning of the magnetic separation seems to be almost independent of the adsorbed layer thickness. This is explained by the fact that only a small portion of nanoparticles composes bulk aggregates, while the main part of nanoparticles forms chains whose capture efficiency is independent of the adsorbed layer thickness but depends solely on the Mason number *Ma*. More precisely, the capture efficiency shows a power law trend $\Lambda \propto Ma^{-n}$, with *n* ≈ 1.4–1.7 at 300 < *Ma* < 10^4^, in agreement with a new theoretical model. Besides these fundamental issues, the current work shows that the multicore IONPs with a size of about 30 nm have a good potential for use in biomedical sensor applications where an efficient low-field magnetic separation is required. In these applications, the nanoparticle surface design should be carried out in a close feedback with the magnetic separation study in order to find a compromise between biological functionalities of the adsorbed molecular layer and magnetic separation efficiency.

## 1. Introduction

Since the 90s, functionalized magnetic nanoparticles are gaining a growing interest related to their biomedical applications, such as magnetic resonance imaging [1,2], magnetic hyperthermia [3,4], drug delivery and release [5,6], mechanical lysis of tumor cells [7,8], protein purification [9,10], microfluidic immunoassays [11], and magnetically tunable scaffolds for tissue engineering [12,13].

Most of these applications require manipulation of magnetic nanoparticles by magnetic field gradients. Because of strong Brownian motion, high magnetic field gradients and strong magnetic fields are often employed for efficient manipulation of magnetic nanoparticles, such as by magnetic sedimentation whereby measuring the concentration decay profile with height above the magnet can lead to the distribution of diameters [14]. Realization of such magnetic circuits is done either by cumbersome and energy consuming electromagnets [15] or sophisticated and presumably high-cost permanent micro-magnets [16]. Nevertheless, despite Brownian motion, it is still possible to get a strong magnetic response of nanoparticles at magnetic flux densities as low as 5–10 mT if they undergo a field-induced phase transition, i.e., gather into elongated bulk micron sized aggregates extended along the direction of the applied magnetic field [17,18], or form fractal clusters under the combined action of colloidal and magnetic dipolar interactions, as inferred from the static light scattering experiments on magnetic liposomes [19]. In the presence of a magnetic field gradient, the aggregates migrate along the field gradient with a speed proportional to the aggregate volume. Such enhancement of the magnetic response is referred to as cooperative magnetophoresis and was studied in detail for magnetic microbeads in Ref. [20,21,22] and for magnetic nanoclusters of an average size of 50 nm in Ref. [23,24].

In particular, the use of cooperative magnetophoresis is expected to be a groundbreaking solution for low field microfluidic magneto-immunoassays based on nanoparticles. Replacement of currently used microbeads by magnetic nanoparticles would allow a considerable decrease of the immunoassay duration due to higher diffusivity [25], together with increase of its sensitivity. However, successful realization of immunoassays (or any other application benefiting from cooperative magnetophoresis) imposes the following requirements to magnetic nanoparticles: (a) they have to possess a relatively strong magnetic moment to be able to undergo a phase transition at low-to-moderate magnetic fields; (b) they have to be superparamagnetic, i.e., their magnetic moment has to undergo thermal fluctuations within the particles to avoid any magnetic dipolar interaction between particles in the absence of an applied magnetic field; (c) they have to possess a narrow size distribution to avoid significant differences in capture efficiency of particle fractions of different sizes; (d) they must bear necessary chemical groups allowing efficient target biomolecule (e.g., antigen) binding and detection; these groups must neither perturb nanoparticle colloidal stability, nor affect their manipulation or separation by magnetic field gradients. Single domain magnetic nanoparticles, like the most widely used iron oxide nanoparticles (IONPs), cannot simultaneously satisfy the requirements (a) and (b) because at sizes below 10–18 nm they are superparamagnetic but are still too small for efficient low field magnetic separation [26], while above 18–20 nm they become ferri- or ferromagnetic [27,28]. Nanoclusters composed of individual nanoparticles associated by either a block co-polymer glue [29] or polyelectrolyte multilayers around a template particle [30], or by a second layer of oleate surfactant [23] respond to the criteria (a) and (b) but are quite polydisperse and may not be suitable for all biological applications.

As an alternative to nanoclusters, several authors [31,32,33,34] have recently synthesized multicore IONPs using a polyol method initially described by Carantu et al. [35]. Among the different reported nanoflowers, the nanoparticles by Hemery et al. [36] are composed of sintered iron oxide cores, each of 7–13 nm in diameter, and have a low polydispersity with the average outer size tuned between 21 and 60 nm. They show high magnetic properties (specific magnetizations close to those of the bulk maghemite and magnetic susceptibility per particle *χ* up to 100) and are superparamagnetic despite their relatively large size. More recently, the same authors have managed to stabilize their multicore particles in water thank to polyethylene glycol (PEG) molecules with phosphonate anchoring groups; they internalized their IONPs into cancer cells and provoked the cell death by heating nanoparticles with an alternating radiofrequency magnetic field (so-called magnetic hyperthermia) [37].

To extend the use of these particles to microfluidic immunoassays, it is important to establish their response towards field-induced aggregation and magnetic separation at microfluidic scale. Thus, the present paper is aimed at establishing peculiarities of kinetics of aggregation of multicore IONPs in the presence of an external magnetic field and of the magnetic separation in a microfluidic channel equipped with a single magnetizable micropillar. Both studies will allow us to set the timescales of aggregate growth and of magnetic separation at realistic magnetic field intensities and flow rates inherent to microfluidic immunoassays. Comparison of these timescales will inform us whether the field-induced aggregation is fast enough to enhance the efficiency of magnetic separation. To assess realistic behaviors in conditions close to immunoassays, the multicore IONPs will be covered with biocompatible PEG molecules at molar mass 6000 g/mol and grafting densities corresponding to the repulsive polymer brush regime [37]. The effect of the grafted layer thickness will be inspected by comparing performances of PEGylated multicore IONPs with citrated ones, citrates being the most common ligand used in the literature to disperse nanoflowers in water [38,39]. The PEG and citrate molecules have rather different sizes but, as will be checked in Section 2.4, both are expected to provide efficient repulsion between nanoparticles in the absence of the magnetic field, such that the chemical nature of the surface layer is expected to play a secondary role as compared to its thickness.

## 2. Materials, Methods, and Particle Characterization

### 2.1. Nanoparticle Synthesis and Functionalization

Multicore IONPs, also called “nanoflowers”, were synthesized by a forced hydrolysis method in polyols, firstly described by Carantu et al. [35], using a modified protocol developed in detail in a previous work [36]. Briefly, ferric and ferrous chloride precursors were dissolved in 1:1 mixture of polyol and poly(hydroxy)amine solvents, namely diethyleneglycol (DEG) and *N*-methyldiethenaolamine (NMDEA). Then they were mixed with stoichiometric amounts of sodium hydroxide and water, and the reactants were heated up at reflux (220 °C) during 5 h. After oxidation into maghemite by treatment with boiling Fe(NO_3_)_3_ [40] and several washing steps, the synthesized nanoparticles were dispersed in water upon grafting of either citrate ions (sample Cit) or PEG with phosphonate anchoring groups (sample PEG). In the case of the citrate ions grafting, 0.2 molar equivalents (relatively to total iron) of tri-sodium citrate salt (Sigma Aldrich, St. Quentin Fallavier, France) was dissolved in MilliQ water and the solution was added at 60 °C under vigorous stirring to the aqueous suspension containing bare multicore IONPs. On the other hand, grafting of the PEG layer was realized using the grafting-to protocol described in detail in Ref. [37]. Firstly, polyethylene glycol methyl ether thiol (mPEG-SH, molecular weight 6000 g/mol, Sigma Aldrich, St. Quentin Fallavier, France) was coupled to 2-aminoethylphosphonic acid (AEP, Sigma Aldrich, St. Quentin Fallavier, France) through the bifunctional *N*-succinimidyl 4-maleimidobutyrate (GMBS, TCI Europe, Paris, France) linker. Then, 12% *w*/*w* (relatively to iron oxide) of aqueous solution of the synthesized AEP-GMBS-PEG molecules was added to the aqueous dispersion of bare IONPs (initially at pH~2 in dilute HNO_3_). The amount of grafted PEG molecules was estimated by thermogravimetric analysis (TGA) and was found to be of 0.106 g PEG per g of iron oxide.

Once dispersed in water, the iron oxide mass concentration, $c_{w}$ (in g/cm^3^) in parent suspensions was estimated using UV-visible spectrophotometry of fully dissolved sample in concentrated hydrochloric acid, as described in Ref. [36]. It was then converted to the volume fraction of iron oxide using the relationship $\varphi_{IO}=c_{w}/\rho_{IO}$, where $\rho_{IO}\approx 4.8\;{g/\mathrm{cm}}^{3}$ is the iron oxide (maghemite) density. We found the values $\varphi_{IO}{}^{\mathrm{spectro}}=$ 0.31 ± 0.02 vol.% and 0.13 ± 0.01 vol.% for the Cit and the PEG parent samples, respectively. These values were compared to those obtained by magnetization measurements ($\varphi_{IO}{}^{\mathrm{VSM}}$) and the average value is defined in Section 2.3.

### 2.2. Particle Morphology and Size

**Figure 1:**
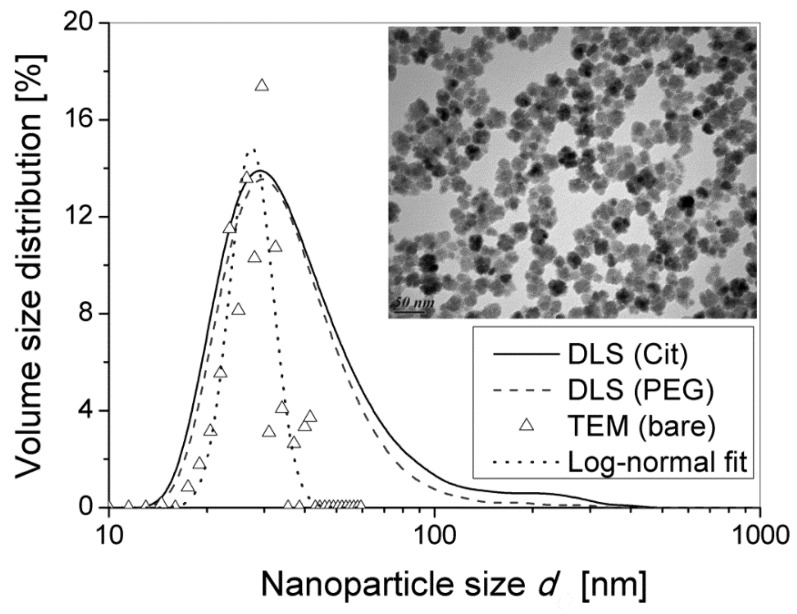
Volume size distribution of multicore iron oxide nanoparticles (IONPs) measured by DLS and TEM. The hydrodynamic size distribution was measured by DLS on IONPs coated by either citrate anions (Cit sample—continuous line) or PEG (PEG sample—dashed line). The TEM size distribution (triangles) was obtained on bare IONPs by analysis of the TEM micrograph shown on the figure inset, followed by conversion of the number size distribution to the volume size distribution for comparison with distributions of the coated IONPs by DLS using Mie theory. The TEM volume size distribution was fitted by log-normal law (dotted line, Equation (1)).

Transmission electron microscopy (TEM) was performed using a Hitachi^TM^ H7650 microscope (Hitachinaka-shi, Japan) with acceleration voltage of 80 kV allowing inspection of the morphology of the as synthesized nanoparticles (i.e., before coating) and dynamic light scattering (DLS, Malvern Zeta Sizer Nano ZS, Malvern, UK) was used to measure the average hydrodynamic diameter *d_O_* of the functionalized nanoparticles. The hydrodynamic volume size distribution (calculated with the Malvern Zetasizer software v. 7.03, Malvern, United Kindom, using Mie theory of light scattering with a refraction index of 2.6 and an absorption coefficient of 0.02 for iron oxide [41] is presented on Figure 1 for both Cit and PEG samples. The DLS distribution width was estimated as $d_{\sigma }\approx d_{O}\sqrt{PDI}$, where *PDI* is the polydispersity index, obtained from the cumulant fit of the DLS correlogram. The volume-average hydrodynamic diameters, the size distribution width and the *PDI* of citrated and PEGylated nanoparticles were $d_{O}\pm d_{\sigma }$ ≈ 45 ± 23 nm, *PDI* ≈ 0.26 and $d_{O}\pm d_{\sigma }$ ≈ 39 ± 20 nm, *PDI* ≈ 0.27, respectively. Since the size intervals of both samples strongly overlap, we cannot conclude on any difference between the hydrodynamic size of citrated and PEGylated IONPs. It is, however, noticeable that the Cit sample contains about 5% of particles with hydrodynamic diameter *d_O_* > 100 nm that likely points out to the presence of transient aggregates in the absence of any magnetic field, as opposed to the PEG sample.

The TEM micrograph of bare nanoparticles is shown on the inset of Figure 1. It was analyzed to yield a histogram of TEM number distribution *f_N_* of diameters *d_I_* of the inorganic multicore. The TEM number size distribution was then converted to the TEM volume distribution *f_V_*, following the formula: $f_{V}(v_{i})=f_{N}(d_{i})v_{i}/\sum f_{N}(d_{i})v_{i}$. Here $f_{N}(d_{i})$ and $f_{V}(v_{i})$ stand for the probabilities that a given particle falls into the size interval $\left[d_{i},d_{i+1}\right]$ or into the volume interval $\left[v_{i},v_{i+1}\right]$, with $v_{i}=d_{i}{}^{3}$ (The numerical multiplier π/6 is omitted before *d_i_*^3^ in the formula for the particle volume *v_i_* because it does not influence the volume size distribution). The obtained TEM volume size distribution was than fitted to the log-normal law that reads as follows:

(1) $$f_{V}(v_{i})=F_{V}(v_{i})\left(v_{i+1}-v_{i}\right)=\frac{1}{\sqrt{2\pi }\beta v_{i}}\exp\left[-\frac{\ln^{2}(v_{i}/d_{\mu }{}^{3})}{2\beta^{2}}\right]\left(v_{i+1}-v_{i}\right),$$

where $F_{V}(v_{i})$ is probability density of finding the particle of a volume $v_{i}$, β = 0.45 and *d_μ_* = 27 nm (median value) are two adjustable parameters. The volume average size *d_I_* with associated standard deviation *d_σ_* are estimated by integration of the log-normal fit distribution, respectively as follows: $d_{I}=\int_{0}^{\infty }v^{1/3}F_{V}(v)dv=d_{\mu }\exp(\beta^{2}/18)$ and $d_{\sigma }{}^{2}=\int_{0}^{\infty }{\left(v^{1/3}-d_{I}\right)}^{2}F_{V}(v)dv=d_{I}{}^{2}[\exp(\beta^{2}/9)-1]$. The experimental TEM volume size distribution and its log-normal fit are shown on Figure 1 by triangles and the dotted line, respectively. TEM confirms the multicore flower-like morphology of synthesized particles with an average grain size of 13 nm and an average multicore IONP size with the distribution width equal to $d_{I}\pm d_{\sigma }$ ≈ 27 ± 4 nm. Recall that such identical bare particles of the inorganic diameter 27 ± 4 nm were covered by either citrate ligands or PEG, and dispersed in distilled water. It is therefore understandable that the volume average hydrodynamic diameters 45 ± 23 nm and 39 ± 20 nm are in average larger than the TEM size and larger than the hydrodynamic diameter of the same batch of multicore IONPs previously reported at 36.5 ± 15.0 nm (*PDI* = 0.16) in the bare state, stabilized by positive charges (protons) at the iron oxide surface in a slightly acidic aqueous medium (HNO_3_ pH~2) [33]. Notice that the size difference between DLS and TEM measurements could also come from some weak aggregation effect of the nanoparticles in the liquid suspensions.

### 2.3. Magnetic Properties

Magnetization *M*(*H*) curve (*M* and *H*—magnetization and magnetic field intensity, respectively) of the acidic aqueous dispersions of uncoated IONPs was measured in our previous work [36]. These measurements showed that saturation magnetization of the solid part of the multicore IONPs was close to that of bulk maghemite (*M_S,IO_* ~ 376 kA/m). Absence of any magnetization hysteresis in the acidic suspension also in the frozen state (268 K) indicates a purely superparamagnetic behavior of the IONPs, meaning that they behave as soft dipoles and that their magnetic moment undergoes thermal fluctuations within the nanoparticles. Otherwise, if they behaved as rigid dipoles, their magnetic moments would be unable to reorient in the absence of Brownian motion in frozen state and to restore zero magnetization at zero applied magnetic field.

**Figure 2:**
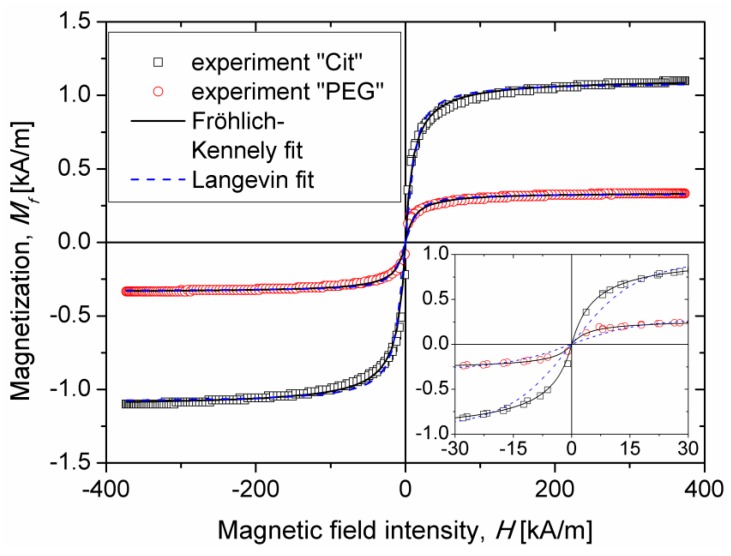
Magnetization curves of aqueous suspensions of multicore IONPs covered by ether citrate anions or PEG. Inset shows a zoomed view of magnetization curves at low magnetic fields. Symbols correspond to experimental data, solid line—Fröhlich–Kennely fit, and dashed line—Langevin fit.

In the present work, magnetization curves of the parent aqueous suspensions of IONPs (coated with citrate anions or PEG) were measured using a vibrating sample magnetometer VSM 4500 (EG&G Princeton applied Research, City, Oak Ridge, TN, USA) at ambient temperature. These curves are shown in Figure 2 for both samples. The Langevin magnetization law widely used for single-core magnetic nanoparticles [27] did not fit satisfactorily to experimental magnetization curves even taking into account particle size distribution (see blue dashed curves on inset of Figure 2). Moreover, the average inorganic diameter of IONPs (without adsorbed surface layer) extracted from Langevin fit was about *d_I_* ≈ 16 ± 3 nm, almost half the diameter *d_I_* ≈ 27 ± 4 nm revealed by TEM. This is likely because the magnetization mechanism of the suspension of multicore IONPs is quite different from that of single-core nanoparticles. It is possible that multicore particles possess a multi-domain structure that could explain their superparamagnetic behavior at the size *d_I_* ≈ 27 ± 4 nm well above the usual critical size corresponding to superparamagnetic-to-ferrimagnetic transition in maghemite or magnetite. Note that establishment of the exact magnetization mechanism was out of the scope of the present paper.

To fit the magnetization curves of IONPs suspensions, we chose the empirical Fröhlich–Kennely law [42], $M_{f}(H)=\chi M_{f,S}H/(M_{f,S}+\chi \left|H\right|)$, that provided a rather good fit with two adjustable parameters: the initial magnetic susceptibility *χ* and the suspension magnetization saturation *M_f,S_*. We got the following values for the parent samples: *χ* = 0.097 and 0.031 and *M_s_* = 1.09 and 0.33 kA/m for the Cit and PEG samples, respectively. Lower value of magnetization saturation of the PEG sample is explained by a lower iron oxide concentration in this sample, as compared to the Cit sample. Iron oxide volume fraction in both parent suspensions was estimated as a ratio of saturation magnetization of the suspension to saturation magnetization of the solid phase of multicore IONPs: $\varphi_{IO}{}^{\mathrm{VSM}}=M_{f,S}/M_{IO,S}$. We obtained the values of $\varphi_{IO}{}^{\mathrm{VSM}}=$0.31 ± 0.02 vol.% and 0.10 ± 0.01 vol.% for the Cit and PEG parent samples respectively. These values are at least rather close to those ($\varphi_{IO}{}^{\mathrm{spectro}}=$0.31 ± 0.02 vol.% and 0.13 ± 0.01 vol.%) estimated by UV-visible spectrophotometry (Section 2.1). The arithmetic mean, $\varphi_{IO}=(\varphi_{IO}{}^{\mathrm{spectro}}+\varphi_{IO}{}^{\mathrm{VSM}})/2$, of both measured values was used as an estimate of the iron oxide volume fraction in both samples, which gives $\varphi_{IO}=$0.31 ± 0.02 vol.% and 0.12 ± 0.02 vol.% for the Cit and PEG parent samples. One can calculate also magnetic susceptibilities per nanoparticle $\chi /\varphi_{IO}$ of 31 ± 3 and 26 ± 5 for the Cit and the PEG samples, respectively, which are outstanding values for superparamagnetic IONPs.

To get finally the volume fraction of multicore IONPs from the iron oxide volume fraction $\varphi_{IO}$, we have to know the internal inorganic volume fraction Ф*_d_* within multicore IONPs, i.e. the ratio of the nanoparticle volume occupied by iron oxide grains to the total nanoparticle volume including the grains and the voids between them. In the literature, the volume fraction Ф*_d_* of analogous structure and sizes than those used in this study was deduced by a nuclear magnetic resonance (NMR) relaxometry study under various fields [43]. Their reported value, Ф*_d_* = 0.72 (or 72 vol.%), will be used in the following calculations, in accordance with helium pycnometry measurements performed on our samples (data not shown). Therefore, the volume fraction of porous multicore IONPs in the parent suspensions is estimated as the ratio of the iron oxide volume fraction to the internal inorganic IONP volume fraction: $\varphi_{0}=\varphi_{IO}/\Phi_{d}$. We obtained the values $\varphi_{0}\approx 0.43\pm 0.03\mathrm{vol}.\%$ and 0.17 ± 0.03 vol.% for the parent Cit and PEG samples, respectively.

The magnetization measurements were necessary to estimate the average magnetic moment, $m_{NP}$ of a single multicore IONP, intervening into calculations of the parameters *ξ* (Equation (7)), *λ* (Equation (8)), and *Ma* (Equation (15)) that govern the kinetics of aggregation and magnetic separation. The parent suspensions satisfy the dilute limit, $\varphi_{IO}<<1$, while the sample vibration during VSM measurements was expected to possibly destroy field-induced nanoparticle structures and restore isotropic spatial distribution of the nanoparticles. At such conditions, the suspension magnetization *M_f_* at each applied magnetic field gives the average magnetic moment, $m_{NP}$, of multicore IONPs when magnetic dipolar interactions between them are negligible, namely $m_{NP}=\mu_{0}M_{f}\pi d_{I}{}^{3}/(6\varphi_{0})$, where $\mu_{0}=4\pi \times 10^{-7}\;H/m$ is the magnetic permeability of vacuum, and the suspension magnetization *M_f_* is calculated for each applied field *H* using the Fröhlich–Kennely law fitted to experimental magnetization curves. It should be pointed out that the value of $m_{NP}$ defined in this way corresponds to the average (over all orientations) of the particle magnetic moment projected onto the direction of the external magnetic field rather than to the saturation value of the magnetic moment.

### 2.4. Samples Dilution and Colloidal Stability

For experiments on the kinetics of aggregation and magnetic separation, the Cit sample was diluted by distilled water to the concentration of the PEG sample and the PEG sample was used as obtained after the functionalization step. More precisely, the iron oxide volume fraction was $\varphi_{IO}$ = 0.12 ± 0.02 vol.%, corresponding to the IONP volume fraction $\varphi_{0}=\varphi_{IO}/\Phi_{d}=0.17\pm 0.03\;\mathrm{vol}.\%$ Two aqueous samples, Cit and PEG, had different pH, ionic strength, and electric charge density on the nanoparticle surface. It was however impossible to adjust the same values of these three parameters that could lead to difference in the colloidal forces between the nanoparticles, according to the standard Derjaguin–Landau–Verwey–Overbeek (DLVO) theory [44]. Nevertheless, such difference might be neglected if the colloidal interactions in both samples were short ranged and non-attractive in the absence of the magnetic field. In such a case, long ranged magnetic dipolar interactions appearing in the presence of a field would be dominant over colloidal ones (van der Waals, steric, and electrostatic forces). The only effect of the adsorbed molecular layer on the IONP surface would be the weakening of the magnetic dipolar interaction parameter $\lambda_{O}$ (Equation (9)) induced by larger separation between IONPs originating from larger PEG chains compared to much smaller citrate molecules. To prove that this condition holds in our samples, we have to estimate the energy of electrostatic and van der Waals interactions between IONPs.

For definiteness, both energies will be estimated at the distance between metal oxide surfaces of multicore IONPs set to the double thickness *h* = 2*δ* of the adsorbed molecular layer. The thickness of the citrate layer *δ*_Cit_ on the surface of Cit nanoparticles is estimated by two different ways: (a) from neutron scattering experiments fitted by Monte Carlo simulations [45], which gives *δ*_Cit_ ≈ 0.2 nm—a value corresponding to the citrate conformation when all the three carboxylate groups are bound to iron oxide surface; (b) assuming that only one carboxylate group is bound to the iron oxide surface while the citrate molecule is stretched perpendicularly to the surface, this gives *δ*_Cit_ ≈ 1 nm. The estimates (a) and (b) provide respectively the lower and the upper bounds of the citrate layer thickness. The thickness *δ*_PEG_ of the PEG layer is more delicate to estimate without knowing the Flory interaction parameter between PEG and water solvent, but the range of *δ*_PEG_ could be obtained by considering two limiting cases of the adsorbed layer morphology: (a) mushroom morphology with the thickness equal to the gyration radius of the PEG chain in water, $\delta_{\mathrm{PEG}}=R_{G}\approx 3.4\;\mathrm{nm}$, as estimated in Ref. [37] for PEG of 6000 g/mol molecular weight; (b) brush morphology with the brush height estimated by Lin and Gast [46] taking into account the grafting density and the curvature of the nanoparticle surface:

(2) $$\delta_{\mathrm{PEG}}\approx {\left[1.025N_{m}{\left(d_{I}/2d_{g}\right)}^{2/3}a^{5/3}+{\left(d_{I}/2\right)}^{5/3}\right]}^{3/5}-d_{I}/2,$$

where *a* = 0.38 nmis the length of the one ethylene glycol monomer, *N*_m_ = 135 is the number of monomers, and *d_g_* ≈ 2.4 nm is the distance between grafting points of PEG brushes on the IONP surface estimated from mass fraction of adsorbed molecules (0.106 g PEG per g of iron oxide as measured by TGA, cf. Section 2.1). The estimation by Equation (2) gives us *δ*_PEG_ = 7.8 nm. Experimentally, the PEG layer thickness can be estimated as a difference of hydrodynamic (measured by DLS) and inorganic (measured by TEM) IONPs radii, which gives us an intermediate value $\delta_{\mathrm{PEG}}=(d_{O}-d_{I})/2\approx 6.0\;\mathrm{nm}$. These three values, *δ*_PEG_ = 3.4, 6.0, and 7.8 nm, will be used below for estimations of the colloidal interactions. 

The energy of van der Waals interaction is estimated in the short distance limit *h* = 2*δ* << *d_I_* [47]:

(3) $$U_{vdW}\approx -\Phi_{d}{}^{2}\frac{A_{H}d_{I}}{24h}\;$$

where *A_H_* ≈ 3.3 × 10^−20^ J is the Hamaker constant for the iron oxide–water pair [48] and the factor Ф*_d_*^2^ (internal particle volume fraction squared) takes into account the fact that the strength of interactions is proportional to the product of numbers of atoms belonging to two interacting particles.

The strength of electrostatic interaction depends on the Debye length $\kappa^{-1}$ and on the particle surface charge density *q*. The first quantity $\kappa^{-1}$ is defined through the ionic strength *I* of the solvent, which in its turn is estimated through the sample electrical conductivity and pH, as described in detail in Ref. [49]. The second quantity, *q*, is deduced from the electrophoretic mobility $\mu_{E}$ measurements carried out using Malver Zeta Sizer Nano ZS. In the present case of thin electric double layer, $\kappa d_{I}>>1$, and moderate surface charge, $eq/(\varepsilon_{0}\varepsilon_{r}\kappa k_{B}T)<2$ the following relationship between $\mu_{E}$ and *q* applies [44]: $\mu_{E}=q\kappa^{-1}/\eta$, where $\eta \approx 10^{-3}\;\mathrm{Pa}\times s$ is the solvent (water) viscosity, $\varepsilon_{0}\approx 8.85\times 10^{-12}\;F/m$ is the dielectric permittivity of vacuum, $\varepsilon_{r}\approx 80$ is the solvent relative dielectric permittivity, $e\approx 1.6\times 10^{-19}\;C$ is the elementary charge. Once the values of $\kappa^{-1}$ and *q* are evaluated, the energy of electrostatic interaction between IONPs is estimated in the short distance limit *h* << *d_I_* and the constant charge approximation as follows [44]:

(4) $$U_{el}\approx -\frac{\pi d_{I}q^{2}\kappa^{-2}}{\varepsilon_{0}\varepsilon_{r}}\ln\left(1-e^{-\kappa h}\right)\;$$

The values of both energies of interaction were evaluated in units of the thermal agitation energy $k_{B}T\approx 4\times 10^{-21}\;J$ at 300 K and are summarized in Table 1 along with the main physicochemical parameters. As inferred from this table, the van der Waals attraction energy ranges between about 2 and 12 *k_B_T* for citrated particles. The electrostatic repulsion energy goes up to 8–24 *k_B_T* at the separation between IONPs equal to double thickness of citrate ions. The resultant interaction (van der Waals + electrostatic) appears to be repulsive for both the lower and upper bounds of the citrate layer thickness. The strength of the resultant interaction ranges between 5 *k_B_T* and 14 *k_B_T* and is expected to be sufficient to ensure good colloidal stability. A small quantity of aggregates of citrated IONPs revealed by DLS (Figure 1) appears likely because small citrate anions are unable to perfectly separate all initially agglomerated particles. In the literature, the stabilizing mechanism of the citrate coating has been shown to be a combination of steric and electrostatic repulsion [50]. It cannot be steric only (as in the case of the neutral PEG coating), as the thickness of the monolayer wrapping the IONPs varies between 0.2 and 1 nm, which in theory is not sufficient to bring a pure steric barrier against van der Waals and magnetic dipolar attractions. Other authors have reported the coating of nanoflowers by adsorbing chains of polyacrylic acid, which is another good stabilizer of IONPs in water [45,51,52].

On the other hand, PEGylated IONPs exhibit both interaction energies below 1*k_B_T* for the three considered values of the inter-particle separation *h* = 6.8–15.6 nm. This means that Brownian motion disperses the PEGylated IONPs while the colloidal interactions may only become important when the adsorbed PEG layers interpenetrate, which causes a strong repulsive barrier [53]. Moreover, since the Debye length is small with respect to particle diameter for both samples, colloidal interactions could be considered as short ranged and should not alter the phase behavior, the kinetics of aggregation and the magnetic separation process in the presence of long ranged magnetic forces, as long as colloidal interactions are represented by hard-sphere potential [54].

### 2.5. Fabrication of Microfluidic Channels

Poly(dimethyl siloxane) (PDMS) cells for the study of kinetics of aggregation (first cell) and of magnetic separation (second cell) were fabricated by soft photolithography. Fabrication of both cells was essentially similar with the difference that the second cell contained a magnetizable micropillar oriented perpendicularly to the cell walls. Thus, in what follows, we describe the fabrication procedure of the second (magnetic separation) cell, bearing in mind that the steps related to realization of the micropillar have to be removed in the description of the fabrication of the first cell.

**Figure 3:**
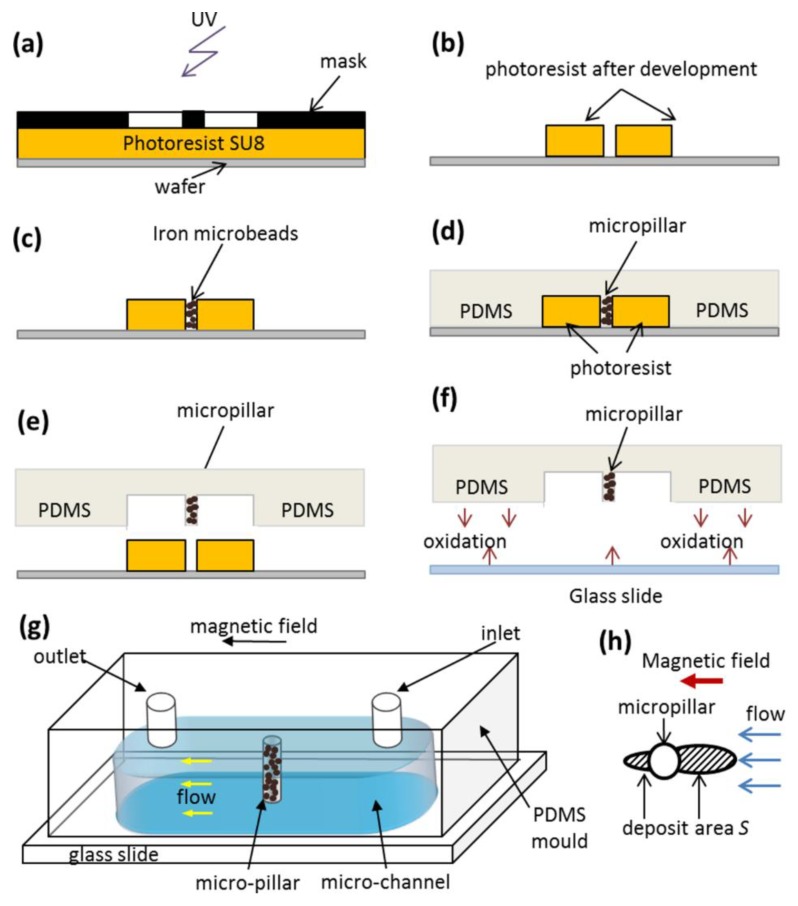
Principle stages of microfabrication of a microfluidic channel for magnetic separation (**a**–**f**). The sketch of the microfluidic channel is presented in (**g**). The deposits of IONPs around the micropillar is presented schematically in (**h**); the deposit surface area *S* is hatched.

The fabrication steps are schematized in Figure 3 and can be summarized as follows. At first, SU8-2025 negative photoresist was spread on a plasma activated silicon wafer using a spin coater (Laurell Technologies, North Wales, PA, USA). The layer thickness was adjusted by rotation speed to 20 and 50 µm for the first and the second cells, respectively. The photoresist was pre-annealed (pre-bake) by putting the wafer on a heating plate at 65 °C and then 95 °C. Then, the photoresist layer was exposed for 4 s to ultraviolet (UV) irradiation through a mask containing a drawing of future microfluidic channel with (2nd cell) or without (1st cell) a circular micropillar. After the post bake, the photoresist was developed with propylene glycol monomethyl ether acetate (PGEMA). The regions irradiated by UV (channel shape) became insoluble and remained on the wafer while the rest of the photoresist was removed. For the second cell, unremoved photoresist contained a cylindrical hole that served as a mold for the future micropillar. This hole was filled with carbonyl iron microbeads (HQ grade, BASF, Schwarzheide, Germany, average diameter about 1–3 µm) by spreading them on the photoresist surface and pushing them into the hole using a brush. The microbeads remaining on the photoresist surface were gently removed by a compressed air jet. To fabricate the channel, PDMS polymer (Sylgard™ 184, Dow Corning, Midland, MI, USA, mixed with its cross-linker at 10:1 proportion and degassed), was poured onto the wafer containing undeveloped resin. PDMS was not cured immediately but was kept at rest for 2 h at 4 °C. During this period, the cavity was completely filled with iron particles and the air bubbles escaped from the cavity thanks to the buoyant force. Then PDMS was cured for 2 h at 50 °C. Finally, the PDMS mold, containing the cavity of desired depth (20 or 50 µm) with or without cylindrical micropillar and embedded iron microbeads, was detached from the wafer. Holes at the entry/exit of the channel were drilled with a punch needle used for biopsy. In order to close the channel, the PDMS piece was glued onto a glass slide after a preliminary oxygen plasma treatment of surfaces. Finally, aluminum needles were introduced into both holes at the extremities of the channel to form the inlet and the outlet. These needles were connected to plastic flexible tubing of a desired diameter serving for connection of the cell inlet with a syringe and as a drainage tube at the cell outlet. A sketch of the fabricated microfluidic channel is presented on Figure 3g. The channels have a length of about 10 mm, width of 1 mm, thickness of 20 µm (1st cell) or 50 µm (2nd cell). The 2nd cell has a cylindrical micropillar of diameter *d_m_* = 50 µm spanning the whole channel thickness.

### 2.6. Experimental Procedure

The experiments on kinetics of aggregation were realized using the following protocol described in detail in Ref. [55]. Suspensions of either citrated or PEGylated IONPs at volume fraction of particles equal to *ϕ*_0_ = 0.17 ± 0.03 vol.% were injected into the first experimental cell using an appropriate syringe connected to the cell inlet. The cell was then placed onto the stage of a transmitted light inverted microscope Daphot (Nikon, Ohi Japan). The external magnetic field (uniform on the cell level) with intensity *H*_0_ = 13.5 kA/m was applied using a pair of Helmholtz coils placed around the microscope and bearing iron yokes. Once the magnetic field was on, the IONPs started to attract each other and form needle-like aggregates which became visible under the microscope several seconds upon the field application. Images of the aggregation process were recorded every minute using a four-fold magnification objective and a complementary metal oxide semiconductor (CMOS) detector camera PL-B742U (PixelLink, Ottawa, ON, Canada) adjusted to the microscope. The images were then processed using ImageJ software (https://imagej.nih.gov/ij/), enabling measurement of the aggregate length profile with elapsed time. The average aggregate length was computed at each moment of time as an arithmetic mean of all the aggregates fitting to the observation window. To check the reproducibility, the measurements were repeated once. The error bars of the aggregate length were calculated as a half of the difference between the values obtained in two measurements.

The experiments on magnetic separation were conducted using the following protocol described in detail in Ref. [56]. The second experimental cell containing a PDMS micropillar with iron microbeads was filled with an aqueous suspension of citrated or PEGylated IONPS at particle volume fraction *ϕ_0_* = 0.17 ± 0.03 vol.% The cell inlet was connected to a 5 mL syringe and the cell was placed on the stage of the same microscope equipped with the Helmholtz coils and with the CMOS camera. The suspension flow at a desired flow rate *Q* through the channel was induced by a syringe pump PHD Ultra (Harvard Apparatus, Holliston, MA, USA). The average velocity, *u* = *Q*/*A* in the channel was tuned in the range 0–10 mm/s, where *A* is the channel cross-section. A few minutes after onset of the flow, the external magnetic field of desired intensity (*H*_0_ = 4.2 or 13.5 kA/m) was applied in the direction parallel to the flow. The micropillar got magnetized and started to capture the IONPs. They accumulated around the micropillar and formed micron sized deposits extended along the applied field. The deposit growth was recorded every minute for 60 min using the four-fold magnification objective and the CMOS camera. The images were then processed using the ImageJ software and the deposit area *S*, schematically shown on Figure 3h was measured at each elapsed time. The relative deposit surface area, *s*, was finally computed as the ratio of the deposit area *S* to the micropillar cross-section: *s* = 4*S*/(*πd_m_*^2^). All experiments were repeated once.

## 3. Results and Discussion

In all experiments, multicore IONPs synthesized by the forced hydrolysis method (Section 2.1) and having the same inorganic diameter (excluding the adsorbed layer) *d*_I_ = 27 nm and the same internal volume fraction Ф*_d_* = 72 vol.% were used. These particles were covered by either a thinner citrate layer (thickness *δ* ≈ 0.2–1 nm—see Section 2.4) or by a thicker PEG layer (thickness ranging between *δ* ≈ 3.4 and 7.8 nm depending on different estimations—see Section 2.4). The outer particle diameter (including the adsorbed layer), was *d*_O_ = *d*_I_ + *h* (where *h* = 2*δ*) and was higher for PEGylated IONPs than for citrated ones.

### 3.1. Kinetics of Aggregation: Microstructure

**Figure 4:**
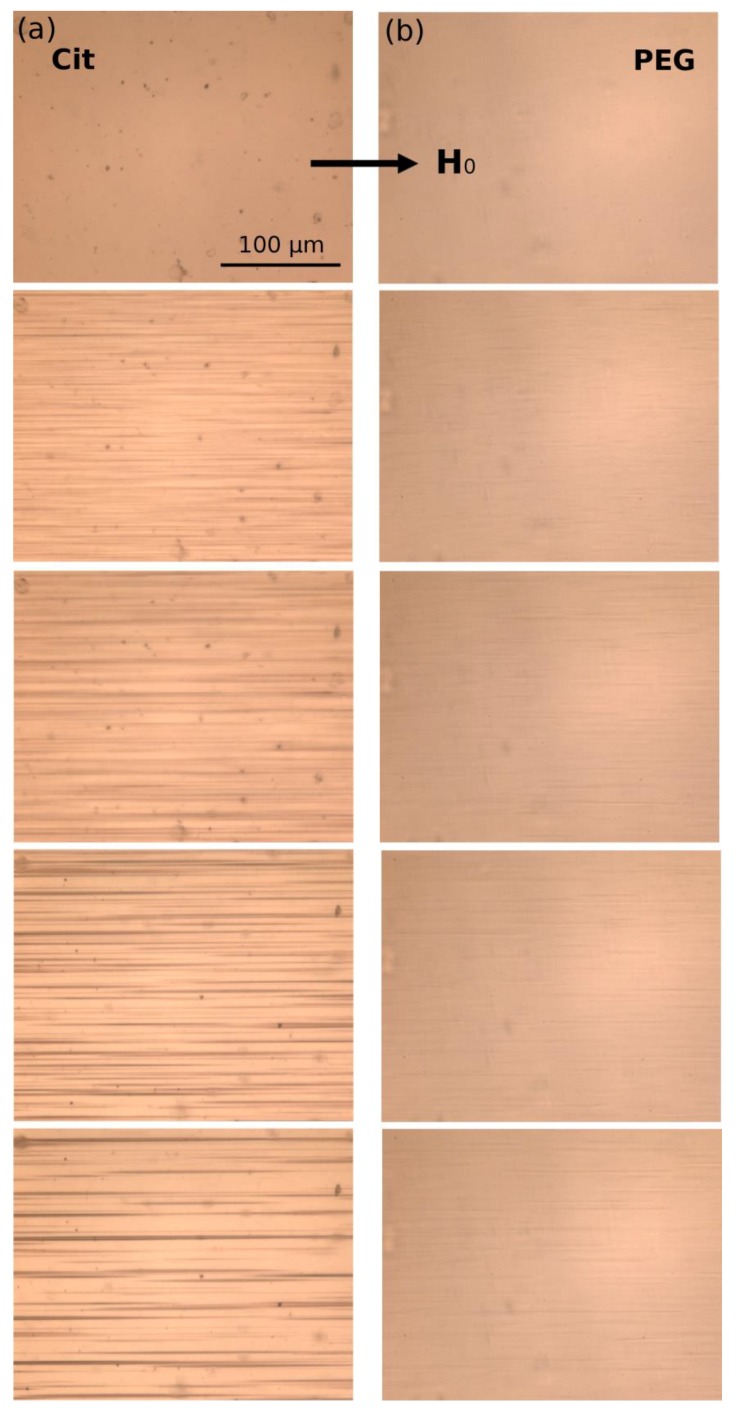
Snapshots of aqueous suspensions of citrated (**a**) and PEGylated (**b**) IONPs at particle volume fraction *ϕ*_0_ = 0.17 ± 0.03 vol.% subjected to an external uniform magnetic field of intensity *H*_0_ = 13.5 kA/m. Each row corresponds to the elapsed time from the moment of the magnetic field application *t* = 0 (upper row), 5, 10, 15 and 20 min (bottom row).

A dilute suspension of citrated (sample Cit) or PEGylated (sample PEG) multicore IONPs at particle volume fraction *ϕ*_0_ = 0.17 ± 0.03 vol.% was introduced in a micron sized channel of thickness 20 µm and observed using an optical microscope. When an external uniform magnetic field was applied, the suspension underwent a reversible phase transition which was recorded with time. The snapshots showing the microstructure of both suspensions at different elapsed times are shown in Figure 4 for an applied magnetic field intensity *H*_0_ = 13.5 kA/m.

The upper row of snapshots corresponds to the elapsed time *t* = 0 at which the magnetic field was just switched on. At this initial moment, both samples did not show any aggregates, however, sample Cit presented some micron-sized particles which could either be dusts or pre-existing agglomerates of IONPs. As the magnetic field was applied, IONPs attracted each other and formed bulk micron-sized needle-like aggregates extended along the magnetic field direction. In the Cit sample, the aggregates clearly appeared around seeds that served as condensation centers for heterogeneous nucleation of IONPs, while these seeds were invisible for the PEG sample. The aggregates grew with time by absorption of neighboring IONPs, and after a certain elapsed time (*t* ≈ 5 min for Cit sample and ≈ 12 min for PEG sample), neighboring aggregates started to coalesce with each other. Thus, the aggregate growth and coalescence stages of particle aggregation were well visible and distinguishable in our experiments. Comparing both samples, one can note much thicker and longer aggregates in the Cit sample than in the PEG sample. This behavior will be inspected in more detail in Section 3.2, where we analyze the aggregate size.

### 3.2. Kinetics of Aggregation: Aggregate Size

**Figure 5:**
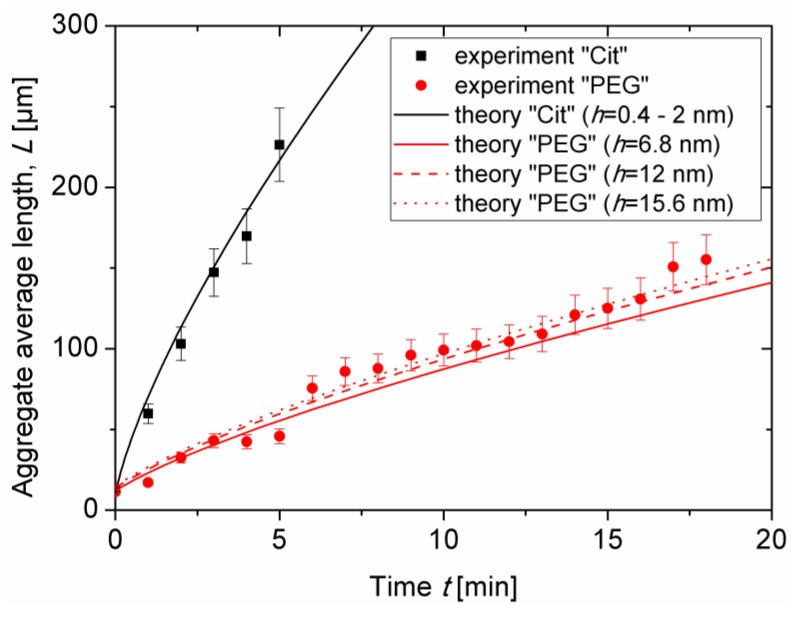
Experimental and theoretical dependencies of the aggregate length on the elapsed time since the beginning of magnetic field application for the citrated and the PEGylated samples respectively, at the same nanoparticle volume fraction *ϕ*_0_ = 0.17 ± 0.03 vol.% and at the intensity of the applied magnetic field *H*_0_ = 13.5 kA/m.

Experimental dependencies of the average aggregate length *L* on elapsed time *t* are presented as symbols in Figure 5 for both samples at the same nanoparticle volume fraction *ϕ*_0_ = 0.17 ± 0.03 vol.% and at an applied magnetic field intensity *H*_0_ = 13.5kA/m. As stated in Section 3.1, the aggregate length continuously grew with time as more and more particles migrated to the aggregates from the surrounding liquid medium. The onset of coalescence of aggregates observed in visualization experiments was accompanied by a slight change of the slope of the experimental *L*(*t*) curves. This break occurred at *t* ≈ 5 min for citrated IONPs and at *t* ≈ 12 min for PEGylated IONPs. Figure 5 also confirms faster aggregation and larger aggregate size for the citrated sample as compared to the PEGylated one. Estimates performed in Section 2.4 have shown that colloidal interactions between IONPs are short-ranged and non-attractive; they are not expected to cause any significant difference in magnetic field-induced aggregation between the two different samples. Thus, from the first glance, the difference in kinetics of Cit and PEG samples is ascribed to weaker magnetic dipolar interactions of PEGylated IONPs covered by a thicker molecular layer than citrated IONPs. For the better understanding of this effect, we proceed to theoretical evaluation of the *L*(*t*) dependency by fitting the experimental results with a theoretical model.

Theoretical *L*(*t*) dependency was derived in a parametric form {*L*(*V*), *t*(*V*)} in our recent work [18] dealing with kinetics of aggregation of intermediate sized (20–100 nm) magnetic nanoparticles, where *V* is the aggregate volume at given time *t*. Despite the onset of aggregate coalescence at *t* ≈ 5 and 12 min for citrated and PEGylated IONPs, respectively, absorption of neighboring nanoparticles by aggregates continued to be the dominant mechanism of aggregation, such that the theoretical model considering only aggregate growth by absorption of individual nanoparticles still provides a rather good fit of the experimental data in the considered range of elapsed time. Adapting the aggregate growth model to the case of IONPs with a nonmagnetic shell of a thickness *δ* = *h*/2 on their surface, the parametric dependency *L*(*t*) takes the following form:

(5) $$t(\hat{V})\approx \frac{K}{10\pi }\left(\frac{B^{2/3}}{D}\right)\frac{{\hat{V}}_{0}}{\Phi_{0}}\frac{{\left(\ln\hat{V}\right)}^{5/7}}{{\hat{V}}^{3/7}}\left[1+0.8{\left(\frac{\Delta }{\Delta_{I}}\right)}^{0.45}{\left(1-\frac{\Delta }{\Delta_{I}}\right)}^{0.3}\right]\ln\frac{\Delta_{I}}{\Delta }\;$$

(6) $$L(\hat{V})=2{\left(\frac{3}{4\pi }\right)}^{1/3}{\hat{V}}^{3/7}{\left(\frac{1}{7}\ln\hat{V}\right)}^{2/7}B^{1/3}\;$$

where $\hat{V}=V/B$ is the dimensionless aggregate volume at given time *t*; ${\hat{V}}_{0}=V_{0}/B$ is the dimensionless volume of primary aggregates at *t* = 0 (the time is counted from the end of very short nucleation stage corresponding to the beginning of the aggregate growth stage); $B\approx \pi^{4}d_{O}{}^{3}/384=\pi^{4}{\left(d_{I}+h\right)}^{3}/384$ is the volume scale; $D=k_{B}T/(3\pi \eta d_{O})=k_{B}T/[3\pi \eta (d_{I}+h)]$ is the nanoparticle diffusion coefficient (as can be assessed by DLS—Section 2.2); $\Phi_{0}$ is the initial volume fraction of aggregates in the suspension; $\Delta \approx \Delta_{0}-\varphi^{\prime \prime }\Phi_{0}\hat{V}/{\hat{V}}_{0}$, $\Delta_{I}=\Delta_{0}-\varphi^{\prime \prime }\Phi_{0}$ and $\Delta_{0}=\varphi_{0}-\varphi^{\prime }$ are the values of suspension supersaturation at given time *t*, at the beginning of the aggregate growth stage and at the beginning of the initial nucleation stage, respectively; $\varphi^{\prime }$ and $\varphi^{\prime \prime }$ are volume fractions of the metal oxide multicores in the suspension corresponding to the left (dilute phase) and the right (concentrated phase) binodal curves of the field-concentration phase diagram. The value $\varphi^{\prime }$ corresponds to the threshold concentration of appearance of aggregates in the suspension at a given field; this value is available experimentally. The value $\varphi^{\prime \prime }$ is close to the volume fraction $\varphi_{d}$ of inorganic multicores inside the aggregates and is estimated assuming random close packing of IONPs within the aggregates taking into account the adsorbed layer thickness: $\varphi^{\prime \prime }\approx \varphi_{d}\approx 0.6{\left(d_{I}/d_{O}\right)}^{3}=0.6{\left(1+h/d_{I}\right)}^{-3}$. Numerical multiplier *K* is a logarithmic function of the Langevin parameter *ξ* and its expression is given in Ref. [18]. The Langevin parameter is a characteristic ratio of the energy of interaction of the IONPs with the external magnetic field of intensity *H*_0_ to the thermal agitation energy *k_B_T* and is defined in the present work as:

(7) $$\xi =\frac{\int_{0}^{H_{0}}m_{NP}dH}{k_{B}T}\approx \frac{m_{NP}H_{0}}{2k_{B}T}\;$$

where $m_{NP}$ is the spatial average (over all possible orientations) of the nanoparticle magnetic moment projected onto the direction of the external magnetic field *H*_0_. This magnitude should not be confused with the saturation value of the magnetic moment and is obtained at each given magnetic field *H*_0_ from magnetization measurements (Section 2.3). Approximate expression in the right-hand side of Equation (7) gives about 10% error in *ξ* at the considered magnetic field intensity *H*_0_ = 13.5 kA/m. Note that the current expression for *ξ* can be reduced to the one used in the original work [18] if $m_{NP}$ is replaced by $3\mu_{0}\beta_{NP}H_{0}V_{NP}=\pi \mu_{0}\beta_{NP}H_{0}d_{I}{}^{3}/2$, where $\beta_{NP}$ and $V_{NP}$ are respectively the magnetic contrast factor and the volume of IONPs. The same remark applies to the definition of the dipolar coupling parameter (Equation (8)) and Mason number (Equation (15)) with respect to the original work [55], except for the fact that Equation (8) should then be multiplied by 4/3.

Theoretical dependencies (Equations (5), (6)) were fitted to the experimental *L*(*t*) curves and are shown in Figure 5 by lines. The best fit was obtained for the following values of two adjustable parameters: ${\hat{V}}_{0}=10^{6}$ and $\Phi_{0}=10^{-9}$. Let us now analyze the effect of the thickness *δ* = *h*/2 of the adsorbed layer on the *L*(*t*)-dependency. Firstly, the Langevin parameter depends only on the inorganic diameter of IONPs, which is the same for both types of nanoparticles; the correction factor *K* is therefore unaffected by the layer thickness. Secondly, the length scale $B^{1/3}$~$d_{O}=d_{I}+h$ and the timescale $B^{2/3}/D$~$d_{O}{}^{3}={\left(d_{I}+h\right)}^{3}$ both increase with the organic layer thickness. Third, the supersaturations *∆* and *∆_I_* decrease with the volume fraction $\varphi^{\prime \prime }\approx \varphi_{d}\approx 0.6{\left(1+h/d_{I}\right)}^{-3}$ of magnetic multicores in the aggregates and increase with the non-magnetic layer thickness *δ* = *h*/2 at the fixed nanoparticle inorganic diameter *d_I_* and at the fixed initial supersaturation *∆*_0_. The net effect of the layer thickness on the *L*(*t*) dependency at fixed *∆*_0_ can be inspected by comparing the solid, dashed, and dotted red lines in Figure 5 corresponding to theoretical evaluation of the aggregate length for the PEG sample at three estimated values (see Section 2.4 for details) of the separation distance between particles: *h* = 2*δ* ≈ 6.8, 12 and 15.6 nm, respectively. The differences between these curves are rather small, so that the effect of the layer thickness on $\varphi^{\prime \prime }\approx \varphi_{d}$ and on the time and length scales causes minor changes on aggregate growth. Notice that two different estimations of the citrate layer thickness (leading to separation distance *h* = 2*δ* ≈ 0.4 and 2 nm) provide hardly distinguishable theoretical dependences *L*(*t*), that are represented by a single black solid line in Figure 5. Fourth, increasing the organic layer thickness is expected to weaken the magnetic dipolar interactions between IONPs and therefore to shift the phase separation to higher particle concentrations at a given magnetic field strength. This effect is reflected in the model by a decrease of the initial supersaturation $\Delta_{0}=\varphi_{0}-\varphi^{\prime }$ with increasing threshold concentration $\varphi^{\prime }$ of the phase separation. The initial supersaturation is the driving force of aggregation, therefore the *L*(*t*) curves are shifted downwards with decreasing *∆*_0_, as the non-magnetic layer thickness increases. The value *∆*_0_ was estimated to be $\Delta_{0}\approx \varphi_{0}=1.7\times 10^{-3}$ for the sample Cit with *h* ≈ 0.4–2 nm and $\Delta_{0}\approx 1.7\times 10^{-4}$ for the sample PEG at *h* ≈ 6.8–15.6 nm. Such a difference in *∆*_0_ values allows reproducing a large difference between experimental *L*(*t*) curves of the citrated and PEGylated samples. It can therefore be concluded that the main effect of the layer thickness on aggregate growth arises from the shift of aggregation threshold to higher particle concentrations as *h* increases.

According to the theory of magnetic field-induced reversible phase separation, the threshold concentration $\varphi^{\prime }$ of the phase separation is a strongly decreasing function of the dipolar coupling parameter [54], which is conventionally defined as the ratio of the dipole interaction energy between two magnetic nanoparticles at contact to the thermal agitation energy [27]:

(8) $$\lambda =\frac{m_{NP}{}^{2}}{4\pi \mu_{0}r^{3}k_{B}T}\;$$

where *r* is the distance between particle centers. One can consider the case when the distance *r* in Equation (8) is set to either the inorganic (*d_I_*) or the outer (*d_O_*) nanoparticle diameter corresponding to two distinct values of the dipolar coupling parameter: $\lambda (r=d_{I})=\lambda_{I}$ and $\lambda (r=d_{O})=\lambda_{O}$. The following obvious relationship between these both parameters holds:

(9) $$\lambda_{O}=\frac{\lambda_{I}}{{\left(1+h/d_{I}\right)}^{3}}\;$$

In the present case of IONPs with an adsorbed surface layer, the parameter *λ_O_* is more appropriate for description of the phase separation, however, evaluation of its value is subjected to uncertainties related to estimating the adsorbed layer thickness *δ* = *h*/2, especially for the PEGylated nanoparticles with *h* ranged between 6.8 and 15.6 nm depending on the conformation of the chains (see Section 2.4). The parameter *λ_I_* is defined with a rather good precision because it depends only on the inorganic nanoparticle diameter defined with confidence from TEM pictures (Section 2.2). This diameter is the same for citrated and PEGylated nanoparticles, therefore, at the same applied magnetic field, the parameter *λ_I_* will take the same value for both types of nanoparticles. The values of *λ_I_* and *λ_O_* evaluated for two intensities *H*_0_ of the applied magnetic field are summarized in Table 2 along with the values of the Langevin parameter (Equation (7)) and of the nanoparticle spatial average magnetic moment $m_{NP}$ calculated from the magnetization curves (Section 2.3). The values of *λ_O_* for citrated and PEGylated particles are shown for respectively two and three different values of the separation between particle inorganic surfaces, estimated in Section 2.4.

As inferred from Table 2, at a given magnetic field intensity, the values of the parameter *λ_I_* are nearly the same for both samples. A slight difference falls within the experimental error of determination by magnetometry of the particle magnetic moment $m_{NP}$. On the contrary, at the same magnetic field, the values of *λ*_O_ are larger by a factor ranging between 1.5 and 4 for the citrated nanoparticles which bear a much thinner citrate layer as compared to the thicker organic layer of the PEGylated nanoparticles. Such a difference in values of *λ_O_* is rather substantial and is expected to be at the origin of the considerable shift of the aggregation threshold to lower particle concentrations as *h* decreases. This suggests that the dipolar coupling parameter *λ_O_* (evaluated taking into account the thickness of the adsorbed layer, Equation (9)) is the major parameter governing the kinetics of nanoparticle aggregation as opposed to the Langevin parameter *ξ* (Equation (7)) giving only a logarithmic increase of the aggregation rate as *ξ* increases. To get explicitly the effect of *λ_O_* on the aggregate growth rate, a *λ_O_*–*ϕ* phase diagram has to be established in future such that the threshold concentration *ϕ*’ should be explicitly expressed through *λ_O_*.

### 3.3. Magnetic Separation: Visualization Results

**Figure 6:**
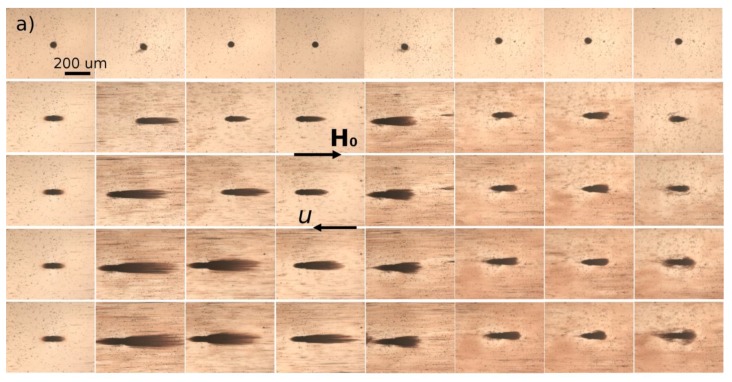

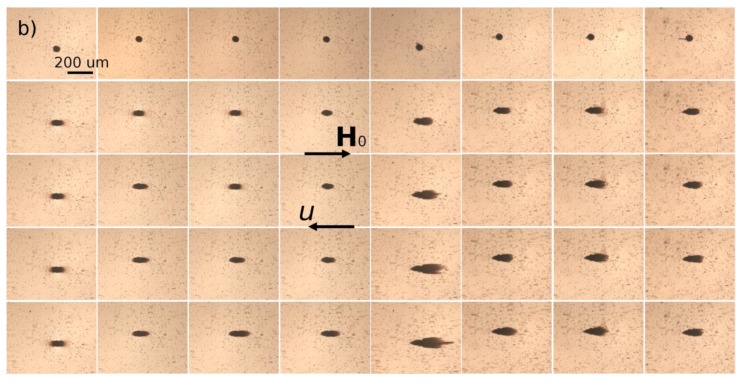
Snapshots of the deposits of citrated (**a**) or PEGylated (**b**) IONPs around a single magnetized micropillar in the microfluidic channel under hydrodynamic flow and externally applied magnetic field of intensity *H*_0_ = 13.5 kA/m at the nanoparticle volume fraction *ϕ*_0_ = 0.17 ± 0.03 vol.% The rows of (**a**) and (**b**) correspond to different elapsed times: from the top to the bottom *t* = 0, 10, 20, 40, and 60 min; the columns correspond to different flow speeds *u* = 0, 0.714, 1.43, 2.14, 2.85, 5.23, 7.61, and 10 mm/s.

We now have to understand the effect of the adsorbed organic layer thickness on the magnetic separation of multicore IONPs. For this purpose, a dilute suspension of citrated or PEGylated IONPs is circulated at a desired flow rate *Q* through a microfluidic PDMS channel containing a single cylindrical micropillar molded in the PDMS with embedded iron microbeads, oriented perpendicularly to the channel walls and spanning the whole channel thickness, as schematically shown in Figure 3g . The micropillar diameter and height are *d_m_* = *h_m_* = 50 µm. Magnetically soft iron microbeads allow reversible magnetization of the micropillar by applying external magnetic fields. When the external magnetic field is applied, either parallel or antiparallel ((with no difference between these two orientations) to the hydrodynamic flow, the micropillar gets magnetized and starts attracting the nanoparticles. The snapshots illustrating accumulation of IONPs around the micropillar under applied magnetic field *H*_0_ = 13.5 kA/m are shown on Figure 6a,b for Cit and PEG samples, respectively. Each column corresponds to different values of the flow speed *u* ranging from 0 to 10 mm/s from the left to the right column. The first row shows the initial state with a homogeneous nanoparticle suspension around a bare micropillar at the moment when the magnetic field is switched on (*t* = 0). Other rows show progressive accumulation of IONPs with the elapsed time ranging from 10 to 60 min from the second to the bottom row.

The nanoparticles of both samples are accumulated around the magnetic poles of the micropillar and over time they form thick deposits extended along the applied magnetic field and having a size comparable and even larger than the micropillar size. At low flow speeds (*u* < 0.714 mm/s and *u* < 2.85 mm/s for the Cit and PEG samples, respectively), the deposits have a rounded shape and a diffuse layer with a smaller particle concentration is observed around their extremities. The diffuse layer disappears at higher speeds and the deposit shape becomes sharper. Spikes on the surface of the rear deposit (situated on the micropillar side opposite to the flow) are detected for the Cit sample at intermediate speeds (0.714 ≤ *u* ≤ 2.85 mm/s). Such behaviors qualitatively agree with those observed for particles deposits around a magnetizable microbead [24] and are interpreted as follows. Since the IONPs suspension has a certain degree of polydispersity (Figure 1), diffuse layer at low speeds likely corresponds to accumulation of smaller IONPs around thick deposits composed of larger particles. This layer provides continuous variation of the magnetic field and of the hydrodynamic pressure on the deposit surface; this favors rounded deposit shapes and excludes surface instabilities. At higher speeds, the diffuse layers are expected to be “squeezed” by the flow, this likely causes discontinuity of the magnetic field and hydrodynamic pressure, which results in sharp deposit shape and possible surface instabilities. It is also remarkable that at all moments of time, the front deposit (facing the flow) is always larger than the rear one. This can be explained by the fact that the front deposit surface is mostly subjected to compressive hydrodynamic force, while the rear deposit is mostly subjected to a tensile hydrodynamic force, so the flux of particles ruptured from the rear deposit is expected to be higher on average than that for the front deposit.

The deposit size shows a non-monotonic dependency on the hydrodynamic flow speed: it increases, reaches a maximum at *u* ≈ 0.714 mm/s and 2.85 mm/s for the Cit and the PEG samples, respectively, and decreases at higher speeds. Such behavior may have different origins for two different samples. Needle-like aggregates were observed in the Cit sample at all speeds except for the quiescent suspension (*u* = 0). At zero and very low flow speeds, the aggregates are expected to settle under gravity and be immobilized at the bottom of the channel before arriving to the micropillar. The aggregates forming in the vicinity of the micropillar were absorbed by the nanoparticle deposits, thus no settled aggregate was observed in the observation window at *u* = 0. We checked that there were a lot of settled aggregates outside the observation window on the left and the right side of the micropillar. At higher speeds, the aggregates were transported by the flow and their fate depended on the compromise between the travel and settling times. Since the aggregates usually had unequal sizes, thicker aggregates settled faster and had less chance to reach the micropillar than thinner ones. With increasing flow speed, the traveling time got shorter and larger aggregates had more chances to touch the micropillar. This explains the growth of the deposit size at low velocity. With a further increase of the speed, hydrodynamic drag forces detaching nanoparticles from the deposit surface increased, and the deposit size decreased.

In addition, the snapshots show that the size of nanoparticle aggregates decreased with increasing flow speed, which could be explained by the fact that the aggregates had less time to grow when traveling from the channel inlet to the micropillar at high speeds. Therefore, the magnetophoretic flux of aggregates is expected to decrease and induce a decrease of the deposit size with increasing flow speed. In fact, the travelling time *t* = *V_s_*/*Q* varies from about 0.5 to 7 min in our experiments, where *Q* is the flowrate and *V_s_* is the suspension volume downstream to the micropillar (including a part of the tubing connecting the syringe pump with the microfluidic channel) exposed to the external magnetic field. During this time, in the quiescent suspension the aggregates achieved sizes ranging between about 50 and 300 µm for citrated IONPs. The size was below 50 µm (the micropillar size) for PEGylated IONPs, as inferred from the study of kinetics of aggregation (Figure 5). As a result, the faster particle aggregation in the Cit sample seems to significantly enhance the accumulation of nanoparticles around the micropillar, as compared to the PEG sample.

In the PEG sample, smaller aggregates were expected to sediment slower, such that the maximum deposit volume was expected at lower hydrodynamic speeds than for the Cit sample, as opposed to what is observed in experiments. However, a smaller magnetophoretic flux of smaller aggregates could result in longer times to build the deposits. It is therefore likely that the steady state was not achieved for the PEG sample at the maximum observation time of 60 min. In other words, the deposits would likely have continued to grow at low speeds and reached their maximum size at *t* > 60 min.

### 3.4. Magnetic Separation: Deposit Growth

**Figure 7:**
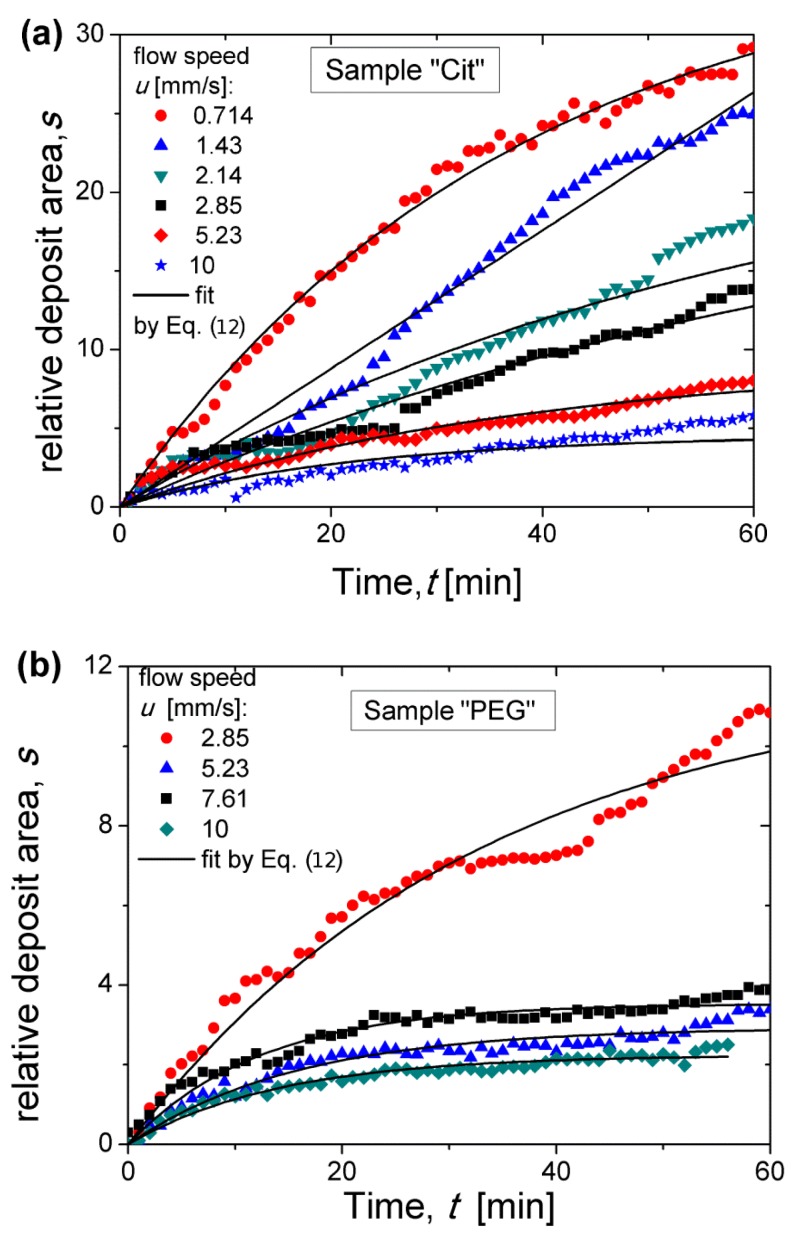
Time dependences of the relative deposit area for the Cit (**a**) and the PEG (**b**) samples at the applied magnetic field intensity *H*_0_ = 13.5 kA/m and for different flow speeds. Symbols correspond to experiments, solid lines correspond to the fit by Equation (12).

To quantify the process of nanoparticle accumulation, the relative deposits area (surface area normalized by the micropillar cross-section *πd*_m_^2^/4) was measured at each elapsed time as described in detail in Section 2.6. Experimental dependencies of the relative deposit area *s* on the elapsed time *t* are shown in Figure 7a,b for the Cit and PEG samples, respectively at different flow speeds. At relatively high flow speeds, the *s*(*t*) curves seem to achieve a final plateau corresponding to the maximum quantity of nanoparticles that the micropillar was able to retain; the value $s(t\to \infty )=s_{m}$ at the plateau is hereinafter called the retention capacity. At relatively low speeds, such a plateau was not achieved and the deposits are expected to grow at *t* > 60 min, as suggested in Section 3.3.

To extract valuable quantitative information from these curves, we have to find an appropriate function fitting the experimental *s*(*t*) curves. A physically relevant fitting function can be found from the balance of the total volume of captured nanoparticles requiring that the growth rate of the deposit area *S* = π*d_m_*^2^*s*/4 is equal to the volume particle flux *J_s_* of particles captured by unit height of the micropillar:

(10) $$\varphi_{d}\frac{\pi d_{m}{}^{2}}{4}\frac{ds}{dt}=J_{s}=J_{0}f(s)\;$$

where $\varphi_{d}\approx 0.6{\left(1+h/d_{I}\right)}^{-3}$ is the volume fraction of inorganic nanoflowers of diameter $d_{I}\approx 27\;\mathrm{nm}$ in the deposit, approximately equal to a random close packing fraction taking into account for the thickness δ=h/2 of the grafted molecular layer on the multicore surface, *J*_0_ is the particle flux at the beginning of the filtration process (*t* = 0), and *f*(*s*) is an empirical function taking into account the decrease of the flux *J*_s_ of captured particles as the deposit area increases. The capture efficiency Λ at the beginning of the magnetic separation is conventionally introduced as the ratio of the particle flux *J*_0_ to the convective flux of particles (by unit micropillar height) through a cross-section whose area is equal to the projected area of the micropillar to the plain perpendicular to the flow [44]. Thus, the expression for the flux *J*_0_ reads:

(11) $$J_{0}=\Lambda \varphi_{0}ud_{m}\;$$

where *ϕ*_0_ is the multicore IONP volume fraction far from the micropillar. It has been checked that the empirical function $f(s)=1-s/s_{m}$, often used in filtration theory [57], ensures a reliable fit of experimental data. Using this function and Equation (11) in Equation (10), we get the following semi-empirical *s*(*t*) dependency:

(12) $$s(t)=s_{m}\left[1-\exp\left(-\frac{4\varphi_{0\;}\Lambda ut}{\pi \;\varphi_{d}s_{m}d_{m}}\right)\right].$$

The experimental *s*(*t*) dependencies were fitted by Equation (12) using two adjustable parameters: the retention capacity *s*_m_ and the capture efficiency Λ related to the initial slope of the *s*(*t*) dependency. Note that the incertitude on Λ includes the error related to determination of the organic layer thickness on the citrated (0.2–1 nm) and PEGylated (3.4–7.8 nm) IONPs and intervening into *ϕ_d_*. This fit is shown by solid curves in Figure 7. As expected, Equation (12) fits the experimental data reasonably well at relatively high speeds when the saturation plateau is well distinguishable. At lower speeds, the experimental curves are far from saturation and the fitted values of the retention capacity *s*_m_ may have not been correctly estimated. The capture efficiency and the retention capacity are analyzed in detail in the two next sections as functions of dimensionless parameters governing the capture process.

### 3.5. Magnetic Separation: Capture Efficiency

First, we intend to predict a scaling law of the capture efficiency as a function of Mason number, which then will be compared to experimental data. Visualization of magnetic separation suggests the existence of nanoparticle aggregates during the magnetic separation process that are well visible in the snapshots of the citrated sample (Figure 6a) but not of PEGylated samples under flow (Figure 6b) (just because they are much thinner and the exposure time of the camera of the microscope was likely insufficient to capture an image of these small objects under flow) but they became detectable if the flow was rapidly stopped. Thus, as the suspension flew along the pathway submitted to the applied magnetic field (including a part of the tubing connecting the syringe pump with the microfluidic channel), the nanoparticles aggregated. According to phase diagrams of dipolar colloids, during aggregation, the bulk needle-like aggregates coexisted with either individual nanoparticles or with single chain-like clusters [58,59]. Therefore, the capture efficiency of such a mixture is the sum of the capture efficiencies of the aggregates (Λ*_a_*) and of the chain-like clusters (Λ*_c_*) weighted by the relative volume of nanoparticles constituting the aggregates and the chains, respectively:

(13) $$\Lambda =\frac{\varphi_{a}}{\varphi_{0}}\Lambda_{a}+\left(1-\frac{\varphi_{a}}{\varphi_{0}}\right)\Lambda_{c},$$

where *ϕ*_0_ is the initial nanoparticle volume fraction in the suspension, $\varphi_{a}=\varphi_{d}\Phi$ is the ratio of the total volume of nanoparticles constituting the aggregates to the suspension volume, Ф is the volume fraction of aggregates in the suspension at a given moment of time, and *ϕ_d_* is the nanoparticle volume fraction inside the aggregates. Note that individual nanoparticles can be regarded as a limiting case of the chain-like clusters with the average number of particles equal to one. 

The capture efficiency of chain-like clusters has been evaluated in our previous work [56]. The chains were supposed to be relatively fragile and ruptured into parts by tensile hydrodynamic forces in the vicinity of the micropillar. The chain length was estimated from the balance of hydrodynamic and magnetic forces exerted to the constitutive nanoparticles. The following scaling relationship for the capture efficiency Λ*_c_* has been found in the limit of high Mason numbers valid in the present work:

(14) $$\Lambda_{c}\propto N^{7/5}Ma^{-1}\propto Ma^{-1.7},$$

(15) $$Ma=\frac{3\pi \eta ud_{I}d_{m}}{m_{NP}H_{0}},$$

where 2 < *N* < 10 is the number of nanoparticles per chain and the Mason number *Ma* is introduced as a characteristic ratio of hydrodynamic to magnetic forces exerted to individual multicore nanoparticles. 

We have now to evaluate the capture efficiency $\Lambda_{a}$ of bulk needle-like aggregates. This magnitude is related to the initial volume flux *J*_0_ of nanoparticles constituting the aggregates (Equation (11)) on the micropillar surface, which is related to the volume flux of aggregates *J_a_* through $J_{0}=\varphi_{d}J_{a}$. Since the aggregates were micron-sized, they were non-Brownian, and on the micropillar surface (where non-slip hydrodynamic boundary condition applied) the only non-zero flux was the magnetophoretic flux whose magnitude by unit micropillar height is given by the following formula [56]:

(16) $$J_{a}=\int_{\Omega }\Phi b_{a}FdP\propto \Phi b_{a}Fd_{m}\;$$

where *b_a_* is the aggregate hydrodynamic mobility perpendicular to the aggregate main axis; *F* is the radial component of the magnetic attraction force between the aggregate and the micropillar, *P*~*d_m_* is the micropillar perimeter; the integration is performed over the part Ω of the micropillar perimeter along which the aggregates are captured. It has been shown that, in the limit of high Mason numbers, Ω tends to *P* and is almost independent of the Mason number [60]. Since the aggregates with a high length-to-diameter ratio, *L*/*d_a_*, are typically observed from the very beginning of the capture process (Figure 6a), the aggregate hydrodynamic mobility scales as $b_{a}$~${\left(\eta L\right)}^{-1}$, neglecting the logarithmic term on *L*/*d_a_* [61]. The absolute value of the magnetic force exerted to the aggregates is $F=m_{a}\left|\nabla H\right|$, with the aggregate magnetic moment estimated as $m_{a}\approx N_{d}m_{NP}$~$Ld_{a}{}^{2}m_{NP}/d_{I}{}^{3}$ and the magnetic field gradient on the micropillar surface scaling as |∇H|~$H_{0}/d_{m}$, where *N_d_* is the average number of nanoparticles constituting the aggregate. This gives us the following scaling law for the magnetic force: F~$m_{NP}H_{0}Ld_{a}{}^{2}/(d_{I}{}^{3}d_{m})$. Combining all the terms together and making use of Equation (11), in which the volume fraction *ϕ*_0_ of all particles has to be replaced by the volume fraction *ϕ_a_* of the particles constituting the aggregates, we get the following scaling relationship for the capture efficiency of the aggregates:

(17) $$\Lambda_{a}=\frac{J_{0}}{\varphi_{a}ud_{m}}\propto {\left(\frac{d_{a}}{d_{I}}\right)}^{2}Ma^{-1}\;$$

Supposing that the aggregate shape is defined by the minimum of its free energy, the aggregate diameter is related to its dimensionless volume $\hat{V}=V/B$ (defined in Section 3.2) through the following approximate relationship [18]:

(18) $$d_{a}\sim B^{1/3}{\hat{V}}^{2/7}\sim d_{I}{\hat{V}}^{2/7}$$

Since the moment when IONPs enter the region of the applied magnetic field, they are expected to form aggregates whose size increases progressively along their pathway before they arrive at the surface of the deposit on the micropillar. Unlike fragile chain-like-clusters, the bulk aggregates are supposed not to be destroyed by hydrodynamic forces in the vicinity of the micropillar provided that their size is comparable to the characteristic length-scale of the velocity variation equal to the micropillar diameter. Thus, their size at the location of the micropillar is defined by the travel time *t* = *V*_s_/*Q* = *L*_c_/*u* corresponding to a characteristic length *L*_c_ of their pathway subjected to the external magnetic field. As mentioned in Section 3.3, the travel time of the nanoparticle aggregates before arriving at the micropillar varies from about 0.5 to 7 min. During this time interval, the experimental *s*(*t*) dependences remain approximately linear, as inferred from Figure 7, such that the travel time can be safely used for evaluating the initial slope of the *s*(*t*) curves related to the capture efficiency. To evaluate the aggregate volume at the time equal to the travel time, we suppose that the aggregate growth in the flowing suspension is descried by the same equation as in the quiescent one, i.e., by Equation (5), which takes the following approximate form (setting $\varphi_{d}$~1 and neglecting logarithmic terms and numerical constants) in the considered interval of travel times 0.5 < *t* < 7 min:

(19) $$\hat{V}\sim \Delta_{I}{\hat{V}}_{m}{}^{3/7}\frac{Dt}{B^{2/3}}\sim \Delta_{I}{\hat{V}}_{m}{}^{3/7}\frac{DL_{c}}{d_{I}{}^{2}u}\sim \Delta_{I}{\hat{V}}_{m}{}^{3/7}\frac{L_{c}d_{m}}{d_{I}{}^{2}}Pe^{-1}$$

(20) $$Pe=\frac{ud_{m}}{D}=2\xi Ma\;$$

where ${\hat{V}}_{m}={\hat{V}}_{0}\Delta_{0}/(\varphi_{d}\Phi_{0})$ is the maximum value of the dimensionless volume of the aggregate growth stage corresponding to the plateau of the $\hat{V}(t)$ curve [18]; $\Delta_{0}$ and $\Delta_{I}$ are supersaturations at the beginning of the nucleation and aggregate growth stages, respectively; the Péclet number *Pe* is introduced as the ratio of characteristic convective to diffusive fluxes of individual nanoparticles and is shown to be proportional to the product of the Langevin parameter (Equation (7)) and the Mason number (Equation (15)). Using Equations (18) and (19) in Equation (17), the expression for the capture efficiency of aggregates takes the final form as follows:

(21) $$\Lambda_{a}\sim {\left(\Delta_{I}{\hat{V}}_{m}{}^{3/7}\frac{L_{c}d_{m}}{d_{I}{}^{2}}\right)}^{4/7}Pe^{-4/7}Ma^{-1}\propto {\left(\frac{\Delta_{I}\Delta_{0}{}^{3/7}}{\xi }\right)}^{4/7}Ma^{-11/7}$$

The mixture rule (Equation (13)) for the capture efficiency of all nanoparticles (belonging to both bulk aggregates and chain-like clusters) gives the Mason number behavior $\Lambda \propto Ma^{-n}$ with the exponent varying from 1.7 (Equation (14)) when all the nanoparticles are gathered into chain-like clusters to 11/7 ≈ 1.57 (Equation (21)) when all the nanoparticles constitute aggregates. The exponent *n* varies therefore in a quite narrow interval 1.57≤n≤1.7 between two limiting cases and we expect the experimental exponent to fall into this interval.

**Figure 8:**
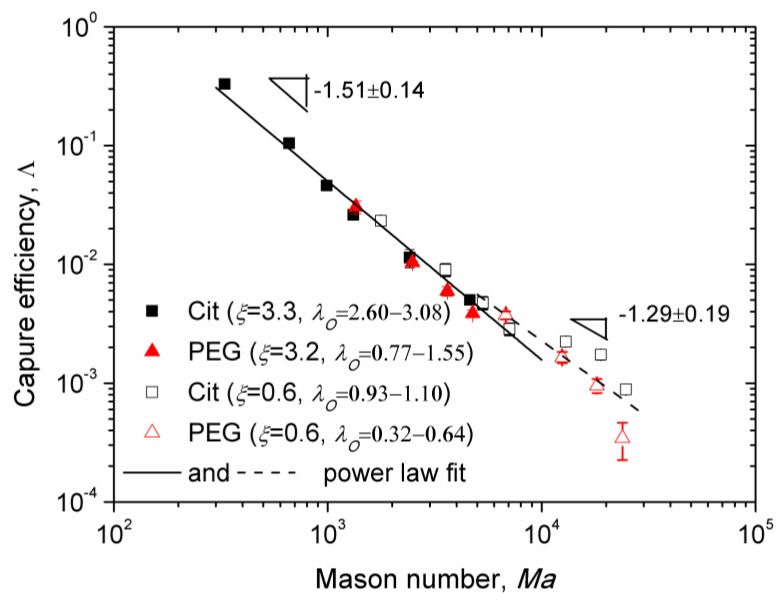
Mason number dependencies of the capture efficiency of citrated and PEGylated nanoparticles at different values of the parameters *ξ* and *λ_O_*. Symbols correspond to experimental points, solid and dashed lines correspond to the power-law fit of the data at, respectively, *Ma* < 10^4^ and *Ma* > 10^4.^

Experimental Mason number dependences of the capture efficiency are shown in Figure 8 for the Cit and the PEG samples for two values of the applied magnetic field corresponding to different values of the parameters *λ_I_*, *λ_O_*, and *ξ*. Here, we excluded the data obtained at low flow speeds for which the majority of nanoparticle aggregates settled on the channel bottom before arriving to the micropillar. All the data for *Ma* < 10^4^ appear to collapse around a single master curve (solid line in Figure 8) showing a power law dependency on Mason number, $\Lambda \propto Ma^{-n}$, with the exponent *n* = 1.51 ± 0.14, whose interval (1.37; 1.65) overlaps with the theoretical interval (1.57; 1.7). At Mason numbers *Ma* > 10^4^, the Mason number effect becomes weaker and the best fit (dashed line in Figure 8) is ensured with a lower absolute value *n* = 1.29 ± 0.19 of the exponent not falling into the predicted interval (1.57; 1.7). This is likely because the flow is too intense at such high Mason numbers and the chain-like clusters are destroyed to individual nanoparticles in the vicinity of the micropillar. The capture efficiency $\Lambda_{NP}$ of individual nanoparticles is obtained by setting *N* = 1 in Equation (14): $\Lambda_{NP}\propto Ma^{-1}$. The bulk needle-like aggregates are still observed at these high Mason numbers, at least for the Cit sample. Thus, the mixture rule (Equation (13)), in which $\Lambda_{c}\propto Ma^{-1.7}$ should be replaced by $\Lambda_{NP}\propto Ma^{-1}$, will give us a theoretical exponent *n* belonging to the interval (1; 1.57). At this condition, the experimental interval (1.10; 1.48), obtained at *Ma* > 10^4^ falls into the theoretical one.

According to our model, the adsorbed layer thickness influences the capture efficiency through the term ${\left(\Delta_{I}\Delta_{0}{}^{3/7}\right)}^{4/7}$, which increases with the dipolar coupling parameter $\lambda_{O}\propto \lambda_{I}{\left(1+h/d_{I}\right)}^{-3}$ and therefore decreases with the adsorbed layer thickness. This seems to be only confirmed for the value $\lambda_{I}\approx 1.2$ (corresponding to *ξ* ≈ 0.6), for which the experimental points of the Cit sample lie in average slightly above the points of the PEG sample. In what concerns the effect of the Langevin parameter *ξ*, according to our model, it affects the capture efficiency through the term ${\left(\Delta_{I}\Delta_{0}{}^{3/7}/\xi \right)}^{4/7}$. However, the experimental data for two rather different values of *ξ* (0.6 and 3.2–3.3) seem to gather along a single master curve whose slope in the log-log scale changes progressively from −1.51 to −1.29 as the Mason number increases. Such data collapse would not occur if the Langevin parameter strongly influenced the capture efficiency.

A small difference between both samples and insignificant effect of the Langevin parameter can be explained by the fact that in the beginning of the intermediate aggregate growth stage of aggregation, only a small portion of nanoparticles composes aggregates, while the rest of the nanoparticles are expected to form chains very quickly during the fast nucleation stage. The capture efficiency of the chain-like clusters does not depend (in the wide *Ma* and *λ_O_* ranges) on *λ_O_* and *ξ* but solely on the Mason number. According to the mixture rule of capture efficiency (Equation (13)), at small fraction of aggregates, $\varphi_{a}/\varphi_{0}<<1$, the contribution of chain-like clusters to the capture efficiency could become dominant and masking the effect of *λ_O_* and *ξ* parameters on the capture efficiency.

Note that the highest capture efficiency Λ≈0.3 has been obtained for the citrated samples at the flow speed *u* = 0.714 mm/s and at a relatively low magnetic field (13.5 kA/m) relevant for microfluidic applications such as magnetic immunoassays [11]. Reliable data for the PEGylated sample has not been obtained for this speed because of unreliable fit of the experimental *s*(*t*) dependencies. However, as Λ(*Ma*) dependencies for PEGylated and citrated samples are almost superimposed on a wide range of Mason numbers (and speeds) as seen on Figure 8, we would expect a nearly similar capture efficiency for the PEGylated sample at *u* = 0.714 mm/s. The value of Λ≈0.3 corresponds to capture of 30% of nanoparticles transported by the suspending liquid through the cross section equal to the projected area of the micropillar. In a realistic microfluidic device, a single micropillar should be replaced by an array of micropillars spanning the entire channel width, thereby significantly increasing the capture efficiency and allowing capturing most of nanoparticles entering the microchannel, as has been recently shown for oleate covered iron oxide nanoclusters [56] of an average size comparable to that of the multicore IONPs used in the current study.

### 3.6. Magnetic Separation: Retention Capacity

**Figure 9:**
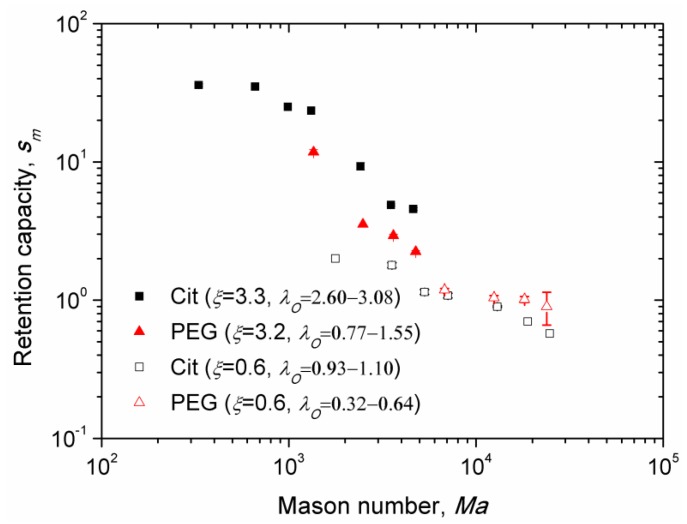
Mason number dependencies of the retention capacity of citrated and PEGylated nanoparticles at different values of the parameters *ξ* and *λ_O_*.

Experimental dependences of the retention capacity versus Mason number are shown on Figure 9 for the Cit and the PEG samples and for different values of the parameters *ξ* and *λ_O_*, recalling that the latter parameter is defined for sample at an uncertainty related to determination of the thickness of the adsorbed citrate and PEG layer (Section 2.4). Similar to Figure 8, we excluded from Figure 9 the data for which the nanoparticle aggregates settled on the channel bottom before arriving to the micropillar. Note that the retention capacity corresponds to the steady-state deposit size at infinite elapsed times. Unlike the Mason number dependency of the capture efficiency, the data of the retention capacity no longer gather onto a single master curve for the PEG and Cit samples. In all cases, the retention capacity is found to progressively decrease with the Mason number, which is confirmed by visualization of the nanoparticle deposits at long elapsed times. At stronger fields *H*_0_ and lower flow speeds *u*, both corresponding to lower Mason numbers $Ma\propto u/(m_{NP}H_{0})$, larger deposits were generally observed (Figure 6).

In the case of the Cit sample, the retention capacity appears to be about five times higher for the Langevin parameter *ξ* = 3.3 than for *ξ* = 0.6 in the range of the Mason numbers 10^3^ < *Ma* < 5 × 10^3^. Furthermore, at nearly the same Langevin parameter, *ξ* = 3.2–3.3, i.e., at the same applied magnetic field, the Cit sample (characterized by the dipolar coupling parameter *λ_O_* = 2.60–3.08) exhibits a retention capacity about twice larger than the PEG sample (with *λ_O_* = 0.77–1.55) in the same range of Mason numbers. These two effects are ascribed to an increase of magnetic interactions between nanoparticles and the micropillar as well as between nanoparticles themselves within the deposit, with increasing *ξ* and *λ_O_* parameters. Stronger magnetic dipolar interactions allow nanoparticles to better resist against rupturing hydrodynamic forces on the deposit surface. Thus, with increasing *ξ* and *λ_O_* parameters, the force equilibrium (defining the location of the deposit surface) was shifted further from the micropillar surface, thereby increasing the deposit volume.

However, the data for the PEG sample at *ξ* = 0.6 does not seem to follow this trend; they show higher retention capacity than for the Cit sample at the same *ξ*. Analyses of the snapshots of magnetic separation of these both samples at *ξ* = 0.6 shows that the deposits of citrated particles were much denser than those of PEGylated ones. This is probably because the magnetic interactions between PEGylated particles (described by the parameter *λ_O_* = 0.32–0.64) are not strong enough and the Brownian motion along with hydrodynamic flow around the micropillar makes the deposits much sparser and more diffuse than the deposits of citrated particles exhibiting a stronger dipolar interaction parameter *λ_O_* = 0.93–1.10. In this case, a clear interface of the deposit does not exist for the PEGylated sample, and the deposit size is rather defined by a characteristic length scale of the concentration variation around the micropillar, which could be larger than the size of the concentrated deposit of the Cit sample. It is very likely that the amount of nanoparticles retained in the dense deposit of the Cit sample is larger than that retained in the sparse deposit of the PEG sample. Unfortunately, we are unable at the present time to evaluate this amount quantitatively.

Finally, it is important to note that at the lowest flow speed (at which nanoparticle aggregates arrive at the micropillar before settling onto the bottom) and at the magnetic field intensity *H*_0_ = 13.5 kA/m, we achieve a steady-state volume of the nanoparticle deposits of about 40 and 10 times of the micropillar volume for the citrated and PEGylated samples respectively. This shows high retention capacity of these nanoparticles at realistic flow rates and relatively low magnetic field, relevant to on-chip applications to immunoassays [11].

## 4. Conclusions

In this work, we have studied field-induced aggregation and magnetic separation of multicore IONPs of an average size of the metal oxide multicore equal to *d_I_* = 27 ± 4 nm and covered by either a citrate or PEG monolayer having a thickness of 0.2–1 nm and 3.4–7.8 nm, respectively. Estimations show that both types of grafted molecules resulted in short-ranged repulsive colloidal interactions between IONPs. In this case, only magnetic interactions between nanoparticles are expected to govern the aggregation and magnetic separation processes. As a consequence, the chemical nature of the grafted layer likely plays a minor role, while its thickness is expected to strongly affect the magnetic dipolar coupling parameter because the organic layer impedes the inorganic cores to be directly in contact together (a situation where the magnetic dipolar attraction forces are maximal). This work was therefore focused on the effect of the grafted layer thickness on the field-induced aggregation and magnetic separation of multicore IONPs with a special accent on enhancement of the separation efficiency by field-induced aggregation. The main results of this work can be summarized as follows:

1. Thinner citrate layer on the IONP surface promotes faster and stronger field-induced aggregation, resulting in longer and thicker bulk needle-like aggregates as compared to those obtained with a thicker PEG layer. This effect is explained by an increase of the aggregation rate thanks to an increase of the suspension initial supersaturation *∆*_0_ as the magnetic interaction dipolar coupling parameter *λ_O_* becomes stronger for IONPs covered with a thinner molecular layer. The experimental dependencies of the average aggregate length versus elapsed time have been satisfactorily fitted to the theoretical model developed in Ref. [18].

2. The magnetic separation of nanoparticles was realized in a microfluidic channel equipped with a single magnetizable micropillar. Measurements of the size of the nanoparticle deposits around micropillars have allowed us to determine the capture efficiency Λ and the retention capacity *s_m_*, both decreasing with increasing Mason number due to the fact that stronger hydrodynamic/weaker magnetic forces hinder attraction of IONPs to the micropillar. Both the theory and experiments confirm the initial hypothesis stating that the capture efficiency is significantly improved if nanoparticles undergo a field-induced aggregation, whose timescale (several minutes) in our experiments was comparable with the travel time of a nanoparticle through the hydraulic circuit of the microfluidic separator.

3. The retention capacity is higher for the IONPs covered by a thinner citrate layer than for IONPs covered by a thicker PEG layer. This phenomenon is attributed to the fact that at the same Mason number, the dipolar coupling parameter of the Cit sample is about 2–3 times higher than for the PEG sample (*λ_O_* = 2.60–3.08 vs. 0.77–1.55). As a result, stronger dipolar magnetic interactions between citrated nanoparticles allow them to better resist against rupturing hydrodynamic forces on the deposit surface, leading to larger deposit sizes.

4. On the contrary, the capture efficiency appears to be almost the same for thick PEG and thin citrate layers on the IONPs surface over the wide range of Mason numbers studied (300 < *Ma* < 10^4^). A small difference between both samples is explained by the fact that only a small portion of nanoparticles composes aggregates. A theoretical model developed in this work predicts a power-law trend $\Lambda \propto Ma^{-n}$ of the Mason number dependency of the capture efficiency with the exponent n∈[1.51,1.7] in agreement with the experimental dependency with n∈[1.37,1.65] at 300 < *Ma* < 10^4^.

Finally, the current work clearly shows that the multicore IONPs of a size about 30 nm that are synthesized by a polyol route have a good potential for use in biomedical sensor applications where an efficient low-field magnetic separation is required. In these applications, the nanoparticle surface design (i.e., choice of grafting density and molecular weight of the adsorbed layer) should be carried out in close feedback with the magnetic separation study in order to find a compromise between biological functionalities of the adsorbed molecular layer and magnetic separation efficiency.

## Figures and Tables

**Table 1:** Interaction energies between multicore IONPs.

Sample	Separation between IONPs *h* = 2*δ* (nm)	Debye Length *κ^−^*^1^ (nm)	Charge Density *q* (mC/m^2^)	Van der Waals Energy |*U_vdW_*|*/k_B_T*	Electrostatic Energy *U_el_/k_B_T*
Cit	0.4	2.3	9.5	11.6	25.4
	2.0			2.3	7.5
PEG	6.8 (mushroom)	4.0	3.4	0.7	0.6
	12.0 (DLS–TEM)			0.4	0.15
	15.6 (brush)			0.3	0.06

**Table 2:** Spatial average magnetic moment of multicore IONPs and parameters *λ_I_*, *λ_O_*, and *ξ* for two values of the applied magnetic field.

Sample	Separation between IONPs, *h* [nm]	*H*_0_ = 4.2 kA/m				*H*_0_ = 13.5 kA/m			
		*m_NP_*× 10^24^ [T × m^3^]	*ξ*	*λ_I_*	*λ_O_*	*m_NP_*× 10^24^ [T × m^3^]	*ξ*	*λ_I_*	*λ_O_*
Cit	0.4 ^(a)^	1.22	0.62	1.15	1.10	2.04	3.33	3.22	3.08
	2.0 ^(b)^				0.93				2.60
PEG	6.8 ^(c)^	1.27	0.64	1.25	0.64	1.98	3.23	3.04	1.55
	12.0 ^(d)^				0.42				1.01
	15.6 ^(e)^				0.32				0.77

^(a)^ estimate from neutron scattering experiments [45]; ^(b)^ maximum size corresponding to fully extended conformation (6 covalent bonds of 0.15 nm giving *δ* ≈ 1 nm); ^(c)^ *h* = 2*R*_G_ (mushroom regime, *R*_G_ is the radius of gyration of the PEG molecule in solution); ^(d)^ *h* = *d*_DLS_-*d*_TEM_ (with *d*_DLS_ and *d*_TEM_—average diameters measured by DLS and TEM, respectively); ^(e)^ brush regime, evaluation by Lin and Gast [46], Equation (2).
